# Process evaluation findings contradict RCT results of the IBD‐BOOST digital self‐management intervention for fatigue, pain and faecal urgency in inflammatory bowel disease: A mixed methods study of patient perspectives

**DOI:** 10.1111/bjhp.70035

**Published:** 2025-11-14

**Authors:** Wladyslawa Czuber‐Dochan, Vari Wileman, Lesley Dibley, Paramasivan Shankavi, Alawi Fatima, Christine Norton, Rona Moss‐Morris, Stephanie J. C. Taylor

**Affiliations:** ^1^ Florence Nightingale Faculty of Nursing, Midwifery and Palliative Care King's College London London UK; ^2^ Department of Psychology Institute of Psychiatry, Psychology and Neuroscience, King's College London London UK; ^3^ Institute for Lifecourse Development, University of Greenwich London UK; ^4^ Wolfson Institute of Population Health Queen Mary University of London London UK

**Keywords:** digital intervention, inflammatory bowel disease, process evaluation, randomised controlled trial, symptom management

## Abstract

**Purpose:**

This parallel process evaluation examined the implementation of a randomised controlled trial (RCT) of IBD‐BOOST—a digital, interactive, facilitator‐supported self‐management intervention targeting fatigue, pain and urgency/faecal incontinence in individuals with inflammatory bowel disease (IBD). The RCT, involving 780 participants, compared IBD‐BOOST with usual care but found no significant differences in quality of life (QoL) or symptom relief at six months post‐randomisation.

**Methods:**

A concurrent mixed methods design was employed. Qualitative data were gathered through semi‐structured interviews; quantitative data were derived from the intervention platform's built‐in analytics. Qualitative data were analysed using narrative thematic and framework analysis; quantitative data were examined using descriptive statistics.

**Results:**

Interviews were conducted with participants pre‐ (*n* = 30) and post‐intervention (*n* = 28). At baseline, participants highlighted a need for improved education and support targeting fatigue, pain and urgency/faecal incontinence, expressing a preference for digital delivery due to its flexibility. Post‐intervention, treatment group interviewees reported high satisfaction with the intervention's content and structure, with many continuing to use the strategies learned, reporting enhanced symptom management and QoL. However, quantitative data indicated low adherence. Control group interviewees expressed disappointment with their allocation but anticipated benefits from deferred access to the intervention.

**Conclusions:**

Although the RCT found no statistically significant effect of IBD‐BOOST on primary outcomes, the process evaluation results revealed perceived benefits in symptom understanding and developing new management strategies. The intervention was well‐received, and patients reported improvements in QoL. There was strong patient support for the IBD‐BOOST intervention to be freely available to all individuals with IBD.

**Statement of contribution**

**What is already known on this subject?**

- Fatigue, pain and urgency/faecal incontinence are common in individuals with IBD, even during remission, and patients report a need for support in managing these symptoms.
- Digital self‐management interventions grounded in cognitive behavioural principles have demonstrated effectiveness in addressing similar symptoms in other long‐term conditions.
- Healthcare professionals, including nurses, are increasingly encouraged to integrate psychological approaches into routine clinical care to support symptom management in IBD.

**What does this study add?**

- Although the IBD‐BOOST cognitive behavioural digital self‐management intervention did not demonstrate effectiveness in the RCT, it was positively received by participants in the process evaluation.
- The intervention improves access to psychological support and aids individuals in managing fatigue, pain and urgency/faecal incontinence.
- Process evaluation findings help contextualise the RCT results, which showed improved disease‐specific QoL at 6 months among those completing four or more of 12 available sessions. Participants advocated for the intervention to be made freely available to all individuals with IBD. Future research should address adherence to enhance effectiveness.

## INTRODUCTION

Inflammatory bowel disease (IBD), encompassing Crohn's disease (CD) and ulcerative colitis (UC), is characterised by relapsing and remitting gastrointestinal inflammation. IBD affects an estimated 6.5 million people globally (Chen et al., 2021) and around 600,000 individuals in the United Kingdom (UK) (IBD UK, 2021). Many patients experience persistent symptoms, including fatigue (47%) (Czuber‐Dochan et al., 2013; D'Silva et al., 2022), abdominal pain (62%) (Lönnfors et al., 2014) and urgency/faecal incontinence (75%) (Norton et al., 2013), even during remission. These symptoms significantly impair quality of life (QoL) (GIBD Collaborators, 2020) and contribute to a substantial economic burden (Burisch et al., 2023). Patients frequently report that their concerns are not sufficiently addressed by healthcare professionals (HCPs) (Czuber‐Dochan et al., 2013, 2014). In a survey of 8486 participants, nearly one‐third expressed a desire for help with all three symptoms (Hart et al., 2024) and in interviews expressed a preference for an online self‐management intervention, citing flexibility and improved control (Fawson et al., 2022).

The online IBD‐BOOST programme (Norton et al., 2021) was developed to address these needs. Informed by cognitive behavioural principles, it comprises 12 sessions: seven core, four symptoms‐specific and one summary session to support sustained improvements (Sweeney et al., 2022). A minimum dose of four sessions were pre‐defined as a pragmatic threshold for meaningful engagement. This decision was informed by prior feasibility work and expert consensus, recognising that participants were expected to focus on those most relevant to their individual symptom management needs. The intervention was co‐designed with patients, feasibility‐tested (Sweeney et al., 2021) and evaluated in a large randomised controlled trial (RCT) (Moss‐Morris et al., 2025; Norton et al., 2021).

Patients were supported by trained facilitators with a 30‐min telephone call (after session 1) and weekly in‐programme messaging during the 12‐week programme. The process evaluation facilitators' findings are presented elsewhere (Wileman et al., Under review). In a trial involving 780 participants, IBD‐BOOST was compared with care as usual. No statistically significant differences in primary outcomes, disease‐specific QoL and global symptom relief, were found at 6 months. However, urgency/faecal incontinence improved in the intervention group, while fatigue and pain did not. Those completing four or more sessions reported improved disease‐specific QoL at 6 months post‐intervention (Moss‐Morris et al., 2025). Process evaluation is an essential component of RCTs, as it helps to understand how complex interventions are implemented and how they achieve their effects. (Moore et al., 2015). Unlike outcome evaluation, it explores mechanisms of impact, fidelity, engagement, adherence and facilitators or barriers to delivery (Durlak & DuPre, 2008; French et al., 2020). It also examines participants' experiences and their influence on outcomes.

By integrating qualitative (e.g., interviews) and quantitative (e.g., adherence rates, usage data) methods, process evaluation yields a comprehensive understanding of intervention delivery and identifies areas for future refinement (Bellg et al., 2004). Particularly valuable in complex interventions, it informs optimisation for future trials or real‐world use (Craig et al., 2008) and supports assessment of long‐term sustainability (Moore et al., 2015). This process evaluation aimed to examine the content and the delivery of the intervention from participants' perspectives to support interpretation of the main trial results and inform future implementation if effective. The study objectives were to:

1. Assess baseline expectations through qualitative interviews.
2. Explore participants' responses to the intervention using both qualitative interviews and quantitative process data.
3. Explore participants' experiences of being in the control group through interviews.
4. Identify potential contextual factors influencing intervention implementation and outcomes.

## METHODS

### Study design and sample size

A concurrent mixed‐methods design was employed. Qualitative data were collected through semi‐structured individual interviews, which explored participants' expectations of, and experiences with, the IBD‐BOOST programme. Quantitative data, such as frequency of platform access, duration of engagement, and the most frequently accessed components, were derived from the platform's built‐in analytics.

Baseline interviews were conducted between January 2020 and May 2022, prior to randomisation, while post‐intervention interviews were undertaken 6 months after randomisation. A sample size of *n* = 30 was deemed sufficient to achieve data saturation (Braun & Clarke, 2022; Busetto et al., 2020). Of the 780 participants randomised in the RCT, 701 (90%) consented to being contacted for a qualitative interview. Participants were invited using a combination of opportunistic and purposive sampling to ensure diversity across trial allocation, site, sex, age and ethnicity. According to the process evaluation log, 36 individuals were initially invited, of whom three declined, one did not respond, and one was pending confirmation. Where an invited participant was unavailable/did not reply (*n* = 2) or declined (*n* = 3) a second interview, a broadly matched replacement was approached. In total, 41 interviews were completed. Of the 28 participants who took part in post‐trial interviews, 15 were in the intervention group and 13 were in the control group.

### IBD‐BOOST intervention content and participants engagement

A total of 780 participants were randomised between 20 January 2020 and 27 July 2022, with 391 allocated to the IBD‐BOOST intervention. Intervention participants were invited to register with the IBD‐BOOST programme, comprising 12 sessions (Table S1). Control group participants received usual care and were offered access to the intervention (without facilitator support) following study completion. Those in the intervention group had 12 weeks' access to the programme supported by a facilitator, followed by continued access with no facilitation for a further 12 weeks, until the primary endpoint at 6 months. Control participants were given access to the intervention, without a facilitator, at 12 months. The intervention's logic model is presented in Figure S1.

### Data collection

Qualitative interviews were conducted via Microsoft Teams©, Zoom© or telephone. Topic guides (Table S2), which varied according to study group (intervention or control) and timepoint (pre‐ or post‐intervention), were developed in line with existing evidence on process evaluation and co‐produced with patient and public involvement and engagement (PPIE) contributors. The interviews explored participants' experiences of living with IBD, their expectations of participation in the IBD‐BOOST programme, and perceived impacts on symptoms such as fatigue, pain and urgency. For example, participants were asked how they managed symptoms before and after the intervention, what aspects of the programme they found most helpful and their views on participating in the control group.

All interviews were conducted by one of two experienced qualitative researchers (W.C.D. and L.D.). Where possible, the same researcher interviewed participants at both timepoints to maintain continuity. Interviews were digitally audio‐recorded and transcribed by a third‐party professional. The following quantitative engagement data from the intervention group were collected via the IBD‐BOOST platform: participants registering on the platform (*n*/%), choice of symptom to focus on (*n*/%), sessions completed (*n*/%), duration of time spent for each session (mean/SD), minimum dose of four sessions (*n*/%), number of tasks completed (*n*/%), facilitator call completed (*n*/%) and mean call duration (min:sec) and participant/facilitator in‐programme messages (mean/SD).

### Data analysis

Reflective thematic analysis was employed to identify and interpret recurring patterns of meaning within the qualitative data, involving familiarisation with the data, generating initial codes, searching for themes, reviewing and refining themes, and defining and naming them. This iterative process allowed for a rich and nuanced understanding of participants' perspectives (Braun & Clarke, 2022). To enhance analytical rigour and reduce researcher bias, transcripts were independently coded by four researchers and eight PPIE contributors. Details of the analytic steps and activities are provided in Table S3.

Data were subsequently subjected to deductive analysis informed by the Analytical Hierarchy Framework (Ritchie et al., 2013) using NVivo 14 software. This involved applying a pre‐defined thematic framework, developed from the initial five transcripts from each dataset. Following the coding of all transcripts, the thematic framework was revised accordingly. The same approach was applied to both baseline and post‐intervention data sets.

Descriptive statistics are presented as mean/standard deviation, median and number/percentage. The online system automatically logged participants' activity. The quantitative analysis was conducted after all participants' access to the platform had ended.

### Ethical considerations

The study received ethical approval from the National Research Ethics Service and Health Research Authority (London – Surrey Research Ethics Committee, reference: 19/LO/0750) and was conducted in adherence with the Declaration of Helsinki (Puri et al., 2009).

Verbal informed consent was obtained and audio‐recorded at the start of each interview. To ensure confidentiality, participants were assigned unique identifiers and all identifiable information was anonymised. Audio recordings were anonymised prior to being transferred to a third‐party transcription service operating under a confidentiality agreement.

A distress protocol was in place, allowing interviews to be paused and participants to be referred to the Crohn's and Colitis UK helpline if required; however, this was not needed. Participants retained the right to withdraw or request deletion of their data within 2 weeks of the interview, although no such requests were made.

## RESULTS

Thirty participants (14M/16F) were interviewed at baseline and 28 (14M/14F) post‐intervention. Of the 28 participants who took part in post‐trial interviews, 15 were in the intervention group and 13 were in the control group. Of the original 30 participants interviewed at baseline, 13 were lost to follow‐up. To address this, 11 additional participants were recruited, resulting in a total of 28 participants completing the post‐intervention interviews. To replace those unavailable or unwilling for a second interview, 11 individuals (demographically matched by age, sex, ethnicity and IBD diagnosis) were recruited for the post‐intervention interviews. Participant demographic and clinical characteristics are summarised in Table 1. Ages ranged from 24 to 76, with most identifying as White British. Interviews lasted between 20 and 76 min, with a mean of 48 min per interview.

**TABLE 1:** Participants' demographic and clinical data.

	RCT sample (*n* = 780)	Qualitative sample (*n* = 41)	Intervention (*n* = 22)	Control (*n* = 19)
Age (mean ± SD)	48.5 ± 14.4	48.8 ± 13.8	50.6 ± 13.6	46.6 ± 14.0
Sex (*n*/%)				
Female	524 (67.2%)	24 (58.5%)	12 (54.6%)	12 (63.2%)
Male	253 (32.4%)	17 (41.5%)	10 (45.4%)	7 (36.8%)
Not stated	3 (0.4%)	–	–	–
Ethnicity (*n*/%)				
White	744 (95.4%)	30 (73.2%)	14 (63.6%)	15 (79.0%)
Non‐white	36 (4.6%)	11 (26.8%)	8 (36.4%)	4 (21.0%)
IBD diagnosis (*n*/%)				
Crohn's disease	432 (55.4%)	23 (56.1%)	15 (68.2%)	8 (42.1%)
UC/other IBD	348 (44.6%)	18 (43.9%)	7 (31.8%)	11 (57.9%)

Themes and sub‐themes identified from baseline and post‐intervention data are outlined in Table 2. These are subsequently discussed with illustrative verbatim quotations (italicised), accompanied by participant ID, sex, age and diagnosis. For brevity, sub‐themes are presented within overarching themes only.

**TABLE 2:** Themes and sub‐themes identified from the interview data.

A. Baseline data		B. Post‐intervention data—intervention group		C. Post‐intervention data—control group	
Themes	Sub‐themes	Themes	Sub‐themes	Themes	Sub‐themes
1. Hopes and expectations from the study	1.1. Improved education about the symptoms 1.2. Learning new methods to manage symptoms 1.3. Preference for allocation to control or intervention group	1. Optimal programme structure and valuable content	1.1. Satisfaction with the programme 1.2. Beneficial content of the programme 1.3. Effectiveness of the programme structure and organisation	1. Expectations of the programme	1.1. Willingness to engage 1.2. Perceived content of the intervention 1.3. Perceived benefits of the intervention
2. Expectations regarding the format and content of the intervention	2.1. Attitudes towards CBT 2.2. Content of the intervention 2.3. Preferred format and engagement methods for the intervention 2.4 Role of the facilitator and communication approach	2. Enhancing symptom control and QoL	2.1. Empowerment through knowledge and improved symptom management 2.2. Benefits of the intervention	2. Motivations and attitudes towards group allocation	2.1. Motivations for programme enrolment 2.2. Attitudes towards the control group allocation
		3. Suggestions for improvement and sustaining success	3.1. Timing of intervention (when it should be offered) 3.2. Enhancing the delivery of the intervention 3.3. Improving and maintaining the programme structure and content 3.4. Importance of the facilitator's role		
		4. Utilisation and engagement with the intervention	4.1. Post‐facilitation (i.e., between 12 and 24 weeks) adoption of the intervention 4.2. Participant involvement and programme completion 4.3. Expectations of the programme		

### Baseline data

Participants experienced a range of persistent and often simultaneous symptoms, including fatigue, pain and urgency/incontinence, that fluctuated in severity and significantly impacted their emotional wellbeing and daily functioning. Despite efforts to identify triggers, many reported limited symptom relief, a lack of control and minimal support from HCPs, reflecting patterns previously documented in the literature.

#### Theme A1: Hopes and expectations from the study

Participants hoped the study would offer practical strategies for symptom management and improve QoL. They valued the study's focus on often‐overlooked symptoms and expressed a desire for a holistic approach to IBD care, incorporating psychological care alongside medical and surgical care.

I really hope that the research gets a positive outcome… getting CBT, getting more than just medical treatment or surgical treatment… maybe psychologists, maybe dieticians. (ID 210174, M, 37, CD)

Some sought to reduce reliance on medication, while others were motivated by a desire to regain energy for everyday activities.

What can I do to help myself and give myself more energy… go and visit someone, do some gardening. (ID 204769, M, 59, CD)

Most participants did not have a strong preference for group allocation, viewing participation as a contribution to research. However, some preferred the intervention group due to anticipated symptom support.

If it's the control one …there's no difference, so for me it would be a bit pointless. (ID 209387, M, 40, CD)

#### Theme A2: Expectations regarding the format and content of the intervention

Most participants viewed a CBT‐based intervention positively, though a few were sceptical due to past negative experiences.

I've had two or three counselling sessions in the past… and I haven't felt any benefit at all. I'm better to go home and sort it out myself. (ID 203866, F, 68, CD)

Many were optimistic about its potential, although some were uncertain about its applicability to physical symptoms like urgency/faecal incontinence.

Participants' interest focused on symptom education, nutrition and exercise, with a preference for individualised advice and practical strategies.

Advice about steps I can take to relieve symptoms… tailored to me as an individual. (ID 209661, M, 29, Other IBD)

Some requested symptom tracking tools and peer support, though concerns about group settings were noted. Most favoured an online format for its flexibility, with some supporting a hybrid model. A weekly time commitment of 1–2 h, combining self‐paced and interactive elements, was considered acceptable.

Many participants expressed a preference for facilitator support, whether through text or phone, valuing guidance on overcoming challenges. They expected facilitators to have expertise in both IBD and general well‐being to provide effective support.

The facilitator should be someone who is both familiar with IBD and knowledgeable on support and wellbeing. (ID 210174, M, 37, CD)

While a few expressed concerns about the programme's demands or effectiveness during symptom flares, most remained committed to completion.

My only concern would be if it's over 12 weeks, it would rather depend on whether I'm having a good week or a bad week… I wouldn't be able to guarantee a certain number of hours, but I would work my way through it. (ID 200133, M, 71, CD)

### Post‐intervention data: Intervention group

#### Theme B1: Optimal programme structure and valuable content

The IBD‐BOOST intervention was perceived to enhance symptom management and QoL. Most participants viewed the programme's structure and content as highly relevant to their needs, particularly appreciating components on stress reduction, sleep hygiene, incontinence management and mindfulness.

I liked the relaxation section; it helped me filter out unnecessary worries. (203641, F, 46, CD)

The programme was especially valued for addressing commonly neglected symptoms such as fatigue. Participants expressed intentions to continue applying the strategies and endorsed wider intervention implementation.

‘I think it's something that should be offered. For me, it was really effective, helpful, and educational’. (203594, F, 29, UC)

I would recommend that anybody has the opportunity to join a BOOST trial or if it gets rolled out permanently, even better. (203641, F, 46, CD)

The digital format was praised for its flexibility, anonymity and accessible presentation (e.g., personalised symptom tracking graphs), enhancing engagement. The timeframe was considered appropriate, with inbuilt breaks supporting reflection.

It was good to have the option to pause the session on a particularly fatigued day. (207012, F, 33, UC)

#### Theme B2: Enhancing symptom control and QoL

Participants reported increased knowledge and coping strategies, notably for stress and incontinence. Many adopted dietary improvements and increased hydration, contributing to improved symptom management.

I've used the information and tools to improve my life and manage my condition better. (207012, F, 33, UC)

Improvements were noted in faecal incontinence, sleep, mental health and overall well‐being. The intervention fostered resilience and positive reframing.

The session helped me control [faecal] incontinence to the point where I no longer consider myself incontinent. (200133, M, 71, CD)

The intervention also encouraged self‐confidence and prioritisation of personal needs.

The course made me think differently about my self‐confidence and what I want out of life. (209474, M, 48, UC)

#### Theme B3: Suggestions for improvement and sustaining success

Several participants recommended offering the programme from age 14 to ideally at diagnosis.

I wish I'd had this when I was first diagnosed. (207012, F, 33, UC)

Some found content emotionally challenging and suggested trigger warnings. Others suggested offline access, clearer upload instructions and resolution of technical issues, including font size.

I experienced technical difficulties, lost notes, and had to redo tasks. (206527, F, 65, CD)

Tailoring content to individual symptoms was frequently requested, with some tasks perceived as irrelevant, but diaries and scenarios were valued. The use of simplified language was suggested.

It was a one‐size‐fits‐all approach. Tailoring it to individual symptoms would be more useful. (200133, M, 71, CD)

Facilitator support was valued but considered insufficient by some. Suggestions included two to three support sessions, instead of just one, spaced throughout the programme.

It was good to talk to someone, but then not having continued contact left me feeling a bit lost. … I expected to talk to someone at the end. (206527, F, 65, CD)

Facilitators played a key role in engagement, with reminders helping participants stay on track.

Once the facilitator's check‐ins stopped, I became less motivated. (209474, M, 48, UC)

#### Theme B4: Utilisation and engagement with the intervention

Many participants adopted the intervention's strategies post‐facilitation period, with approximately three‐quarters continuing their use post‐intervention. There was strong support for incorporating the programme into standard treatment pathways.

I would love to see it integrated into a long‐term treatment approach. (210174, M, 37, CD)

Engagement with the intervention varied, with participants reporting between 30 min to 1.5 h per session. Self‐reported time spent on the platform broadly aligned with usage metrics, lending support to the credibility of participants' accounts. Some completed sessions in one go, while others took breaks or completed them over a few days. Competing demands were cited as barriers to completion. Participants working remotely reported benefiting more from the programme due to reduced travel and increased flexibility. Sharing content with family was suggested to enhance impact.

It would have been useful to share it with my family for additional support. (203963, M, 63, UC)

A few participants were unsure about the programme's purpose, which affected their engagement. While many had no prior expectations, some found the programme more demanding than anticipated.

It was more complicated than I expected in terms of commitment. (206527, F, 65, CD)

### Post‐intervention data—control group

#### Theme C1. Expectations of the programme

This theme explores participants' expectations and initial willingness to engage with the intervention. Most expressed readiness to participate once the programme became available and anticipated setting aside time for the tasks.

Yes, I'm looking forward to it, because it sounds like there might be things out there that can help me with my fatigue to manage it. (208405, F, 51, CD)

Anticipated benefits, particularly symptom improvement, were key motivators.

If it's got benefit or potential benefit to me, I will try it and see it through. (205050, M, 40, CD)

While many correctly anticipated cognitive behavioural therapy (CBT)‐based strategies, there was uncertainty about the specific content.

I'm not sure what the content of the intervention would have been and whether it was advice or whatever. (203409, M, 65, UC)

Participants sought content addressing diet, fatigue, urgency/faecal incontinence and stress, with a preference for individual over group delivery. Expectations aligned with the intervention's intended structure. Anticipated benefits included improved symptom management, enhanced self‐confidence and better QoL.

I hope that it will help in some way and just help everybody feel better with their condition and just be happier. (208225, F, 35, UC)

#### Theme C2. Motivations and attitudes towards group allocation

Motivations for participation ranged from a desire for personal benefit to learn new methods for symptom management, to contributing to research for the wider community.

I took part in it because I thought there would be things that I could help to offer other people. (208405, F, 51, CD)

While those with milder symptoms expressed less urgency, others enrolled in anticipation of their symptoms worsening in the future.

I am not now or when I signed up suffering badly enough with my symptoms that I am desperate for something to help. (202607, M, 40, UC)

Of the 11 control group participants, nine were satisfied with being allocated to the control group, citing the opportunity to access the intervention at later date, while two expressed dissatisfaction.

### Quantitative data on participant engagement with IBD‐BOOST programme

Of the 391 individuals who were randomised to the intervention group, 388 (99%) completed registration and 346 (89%) initiated the programme by completing Session 1; 45 (11.5%) did not complete any sessions. Participants identified their most bothersome symptom to target within the IBD‐BOOST programme: fatigue (*n* = 188, 53%), pain (*n* = 45, 13%) and urgency/faecal incontinence (*n* = 119, 34%). The IBD‐BOOST programme was designed for sequential completion, with core sessions delivered in a fixed order to build on previous content. Participants who completed only one to three sessions consistently engaged with the initial sessions of the programme.

**FIGURE 1:**
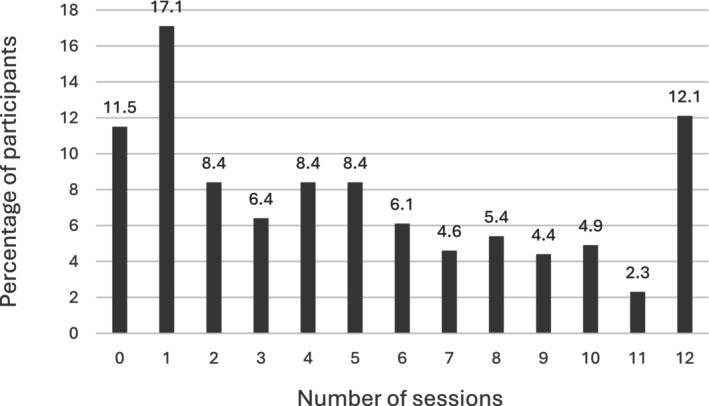
Percentage of participants who completed number of sessions.

The mean number of sessions completed was 5.0 (SD = 4.0; see Figure 1). Sixty‐seven participants (17%) completed only one session, while 47 (12%) completed all 12. In total, 221 participants (57%) met the pre‐defined minimum dose of four sessions.

### Duration of time spent engaging with the IBD‐BOOST programme

Table 3 presents the number of participants who complete each session, and the corresponding time spent. Typically, the time spent was less than the recommended session time. One exception was Session 5: Managing Stress and Coping with Emotions, which had a median duration of 69.5 min.

**TABLE 3:** Participant time spent completing sessions and tasks.

	Session/task topic	Protocol recommended time (min)	Number of participants completed session/task	Duration spent (min)^a^		
				Min	Max	Median
Session 1	Understanding your IBD symptoms	45–60	346	5	235	32
Session 2	Balancing your activity, eating and exercise	45–60	269	6	282	39
Session 3	Improving your sleep	30	232	2	1749	19
Session 4	Changing your thoughts Part 1	30	212	2	146	25
Session 4b	Changing your thoughts Part 2	30	169	1	1149	27
Session 5	Managing stress and coping with emotions	45–60	140	6	364	69.5
Session 6	Making the most of your social support and communication	45–60	118	2	205	29
Session 7	Managing and understanding fatigue in IBD	30–45	103	0	268	28
Session 8	Managing and understanding pain in IBD	30–45	72	1	150	31
Session 9	Managing and understanding urgency and leakage in IBD	45–60	144	2	386	41
Session 10	The role of acceptance and self‐compassion in pain	30–45	68	1	167	24
Session 11	Summary and maintaining improvement	30–45	76	1	175	28
Task 1	Understanding your IBD symptoms		214	0	150	9
Task 2	Balancing your activity, eating and exercise		165	0	224	13
Task 3	Improving your sleep		163	0	93	1
Task 4a	Changing your thoughts Part 1		139	0	146	6
Task 4b	Changing your thoughts Part 2		108	0	95	6.5
Task 5	Managing stress and coping with emotions		83	0	114	5
Task 6	Making the most of your social support and communication		78	0	56	0

*Note*: Data for completed sessions only.

^a^ Time only recorded for in‐programme task.

### Participant interaction with their assigned facilitator

Sixteen trained facilitators supported participants over a 12‐week period, after which programme access continued for an additional 12 weeks without facilitator involvement. All participants who completed Session 1 were offered a 30‐min telephone treatment call. Of the 346 who completed Session 1, 306 (88%) received this call, with an average duration of 34.3 min (SD = 7.5). For approximately 12% of participants, facilitator calls were not completed primarily due to difficulties in contacting the participants and challenges in scheduling a mutually convenient time, despite multiple contact attempts by facilitators.

Facilitators also sent weekly messages via the IBD‐BOOST platform, with high fidelity to the protocol (Wileman et al., Under review). The intervention protocol instructed facilitators to send one supportive message per week to participants to maintain engagement and provide ongoing encouragement. However, the actual number of messages sent varied according to individual participant preference and responsiveness. Some participants required more frequent contact due to questions or difficulties, while others preferred less frequent communication. This flexible approach allowed facilitators to tailor support to maximise participant engagement and address specific concerns as they arose. On average, 16.0 messages (SD = 7.5) were sent per participant. Participants sent a mean of 5.3 messages (SD = 4.3), ranging from 2 to 23.

## DISCUSSION

This process evaluation provides important contextual insights into the delivery of, and participants' perceived value of, the IBD‐BOOST intervention. While the main RCT did not demonstrate statistically significant group‐level effects, participants in this process evaluation consistently reported the intervention as acceptable, relevant and beneficial. These findings highlight the potential for the programme to support individuals with IBD in managing symptoms such as fatigue, pain and urgency/faecal incontinence, areas often under‐recognised in routine care (Czuber‐Dochan et al., 2013, 2014; Dibley et al., 2021; Fawson et al., 2022; Proudfoot et al., 2018; Sweeney et al., 2021).

Participants perceived the intervention as beneficial because it addressed both practical (exercise, diet, sleep hygiene) and psychological aspects (stress reduction, mindfulness, prioritisation) of living with IBD symptoms through a flexible, accessible and structured format. Participants reported that the content was highly relevant to their needs, particularly strategies for managing fatigue, stress, sleep and urgency/faecal incontinence. The integration of cognitive behavioural principles enabled users to reframe negative thought patterns, build self‐efficacy and develop post‐facilitation period coping strategies (Ballou & Keefer, 2017; Emerson et al., 2021; Khera et al., 2019).

The digital format of the intervention was highly valued for its convenience, allowing participants to engage at their own pace and revisit content when needed, which was especially important during symptom flares or fatigue. The perceived anonymity and control over engagement may have further supported honest reflection and sustained use.

Facilitator support, especially the initial telephone call and ongoing in‐programme messaging, was motivating, although some participants expressed a need for more consistent input throughout the programme. Participants also appreciated the educational content and reassurance regarding their symptoms, which contributed to improved emotional wellbeing and a greater sense of control (Sheehan et al., 2023). However, technical issues, perceived lack of personalisation and limited facilitator contact were noted as barriers to engagement. Suggestions for improvement included clearer structure, content tailored to individuals' needs, better digital usability and expanded facilitator involvement to 2–3 sessions throughout the programme.

Despite positive feedback, overall engagement was modest. While 89% completed Session 1, only 57% achieved the predefined minimum dose of four sessions and just 12% completed all 12. Importantly, participants were not expected to complete all 12 sessions, but only the generic sessions and the specific session/s relevant to their primary symptom, indicating the intervention's individualised design. Engagement metrics revealed considerable variation in how participants used the IBD‐BOOST programme. While many completed sessions fully, some accessed content only briefly, with minimum session durations as short as 0–2 min. These brief interactions may reflect participants ‘sampling’ the material rather than fully engaging, which could influence intervention effectiveness. This variability highlights the challenges of measuring adherence in digital self‐management programmes and suggests the need for further exploration of what level of engagement is sufficient to achieve meaningful benefit. Low engagement may reflect time constraints, fluctuating symptom severity and limited perceived relevance. Notably, some participants with mild or well‐controlled symptoms at baseline reported limited engagement but valued retaining the content and learning new coping strategies for future use. The evaluation illustrates that symptom severity may drive engagement, with participants experiencing active symptoms deriving greater benefit. Tailoring recruitment to patients with greater symptom severity and adjusting content accordingly may enhance effectiveness.

Although the IBD‐BOOST programme provided participants with the option to select sessions relevant to their symptoms, qualitative feedback suggested a desire for even greater tailoring of content. To enhance individualisation, future refinements could incorporate increased facilitator involvement to personalise support more dynamically throughout the intervention. Additionally, integrating adaptive digital features, such as algorithm‐driven content recommendations based on participant progress and preferences, may further optimise engagement and relevance. These strategies have the potential to improve both adherence and effectiveness by better addressing the unique needs of each participant and warrant exploration in subsequent iterations of the programme.

Baseline expectations showed strong willingness to engage, driven by a desire to better manage symptoms, particularly fatigue, urgency/faecal incontinence, stress and negative coping. Most anticipated a CBT‐based approach and preferred individual over group delivery, aligning with the intervention design.

Experiences of the control group participants captured through interviews indicated that although two participants expressed dissatisfaction with group allocation, most were content due to the prospect of delayed intervention access. In addition, many participants did not have severe symptoms, which may have reduced the perceived need to be allocated to the intervention group. Motivation for trial participation included both personal benefit (gaining new knowledge and learning new skills to manage the symptoms) and altruistic intentions of contributing to research for the benefit of others. These insights highlight the importance of clearly communicating the trial purpose and design to minimise attrition and dissatisfaction.

Contextual factors, such as symptom severity, competing demands, time required to complete each session and additional tasks, technical challenges and facilitator input, impacted engagement. Although, some participants indicated that they would have preferred more frequent contact with the facilitator to help sustain motivation.

Key components for successful implementation include digital flexibility, personalised symptom targeting and structured facilitator support. Future iterations should refine onboarding, streamline navigation, improve technical access and introduce adaptive pacing. Integration into standard care pathways, supported by HCPs, may improve uptake and scalability. Participants recommended long‐term access to content and optional peer support features, such as moderated forums or shared modules with family, to reduce isolation and enhance engagement.

The intervention's perceived long‐term utility, particularly for under‐managed symptoms, highlights its potential as a sustainable adjunct to clinical care. Embedding the programme within digital health platforms and ensuring alignment with NHS standards would support clinical integration. Use of trained non‐clinical staff could offer scalable delivery while maintaining personalisation.

### Strengths and limitations

This study is strengthened by its parallel mixed‐methods design, enabling triangulation of qualitative interviews with quantitative process data to provide a comprehensive understanding of engagement, intervention response and contextual factors. The large, diverse sample and inclusion of both intervention and control participants enhance the credibility and transferability of findings.

The intervention's flexible, symptom‐focused digital format aligned well with participant needs, and sustained engagement suggests potential for behavioural change and scalability. The process evaluation offers detailed insight into delivery and acceptability, supporting interpretation of trial outcomes and informing future development.

Self‐selection may have introduced bias, favouring more motivated or health‐literate individuals, while voluntary interview participation may have led to overrepresentation of particularly positive or negative views. Some baseline interviewees were unwilling or unavailable for a second post‐trial interview and were replaced by demographically matched interviewees. It is unclear whether or how this may have influenced our qualitative findings. We did not collect information on participants' prior experiences with psychological therapies, which may have influenced their perspectives towards the intervention. Digital literacy and technical issues posed barriers for some and limited content personalisation was noted. These factors may have influenced engagement and reduced transferability.

Despite these limitations, the study offers valuable guidance for optimising digital interventions for people living with IBD.

## CONCLUSION

The success of IBD‐BOOST depends on alignment with patient needs, flexible delivery, consistent support and integration into routine care. Findings indicate the intervention is feasible and acceptable, with digital flexibility, tailored symptom focus and facilitator contact driving engagement. The process evaluation highlights the value of qualitative insights in interpreting trial outcomes and informing future refinement and implementation.

Despite negative RCT results, participants valued the structured, flexible format and symptom‐specific content, particularly strategies addressing fatigue, stress and urgency/faecal incontinence. High initial engagement and continued use suggest potential for lasting behavioural change. While facilitator support was generally well received, more sustained contact may further enhance engagement.

The evaluation also underscores the importance of contextual factors such as symptom profiles, digital literacy and technical accessibility. Increasing the minimum dose may improve efficacy.

Overall, these findings support the feasibility and acceptability of IBD‐BOOST and offer direction for optimising its delivery, impact and scalability.

## AUTHOR CONTRIBUTIONS

**Wladyslawa Czuber‐Dochan:** Conceptualization; methodology; writing – review and editing; data curation; funding acquisition; investigation; visualization; formal analysis; writing – original draft; supervision. **Vari Wileman:** Data curation; investigation; writing – review and editing; writing – original draft; formal analysis; methodology; visualization. **Lesley Dibley:** Conceptualization; funding acquisition; writing – review and editing; investigation; formal analysis; data curation. **Paramasivan Shankavi:** Data curation; investigation; writing – review and editing; formal analysis; visualization. **Alawi Fatima:** Data curation; formal analysis; writing – review and editing; investigation; visualization. **Christine Norton:** Conceptualization; funding acquisition; writing – review and editing; methodology; supervision. **Rona Moss‐Morris:** Conceptualization; funding acquisition; writing – review and editing; methodology. **Stephanie J. C. Taylor:** Conceptualization; funding acquisition; writing – review and editing; supervision.

## FUNDING INFORMATION

The study was funded by the National Institute of Health Research Programme Grant, Reference number: RP‐PG‐0216‐20001. The views expressed in this publication are those of the authors and not necessarily those of the National Institute for Health Research or the Department of Health and Social Care.

## CONFLICT OF INTEREST STATEMENT

WC‐D: Speaker fees from Dr. Falk, Pharmacosmos and research funding from Bristol Myers Squibb and Crohn's and Colitis UK; LD: Speaker fees from Janssen, Tillotts Pharma UK, Ferring Pharmaceuticals; research funding from Janssen and Takeda; CN: Speaker fees from Medscape, Merck Pharmaceutical; Tillotts Pharma UK, Lilly. Pfizer advisory board; RM‐M: A beneficiary of a licence agreement signed between King's College London and Mahana Therapeutics for a digital cognitive behavioural therapy for an irritable bowel syndrome product. RMM receives personal fees from Mahana Therapeutics for scientific advisory work and from other universities and hospital trusts for cognitive behavioural therapy training in irritable bowel syndrome. Other co‐authors declare no competing interests.

## Supporting information


Figure S1.

Table S1.

Table S2.

Table S3.

## Data Availability

The data underlying this article cannot be shared publicly due to the sensitive nature of the research and the privacy of the individuals who participated in this study.
