# Supplementary figures and images for: Process evaluation findings contradict RCT results of the IBD‐BOOST digital self‐management intervention for fatigue, pain and faecal urgency in inflammatory bowel disease: A mixed methods study of patient perspectives
Source: Br J Health Psychol. 2025 Nov 14;30(4):e70035. doi: 10.1111/bjhp.70035 (PMC12617385; doi:10.1111/bjhp.70035)

**Figure S1 Logic model for the IBD-BOOST intervention**


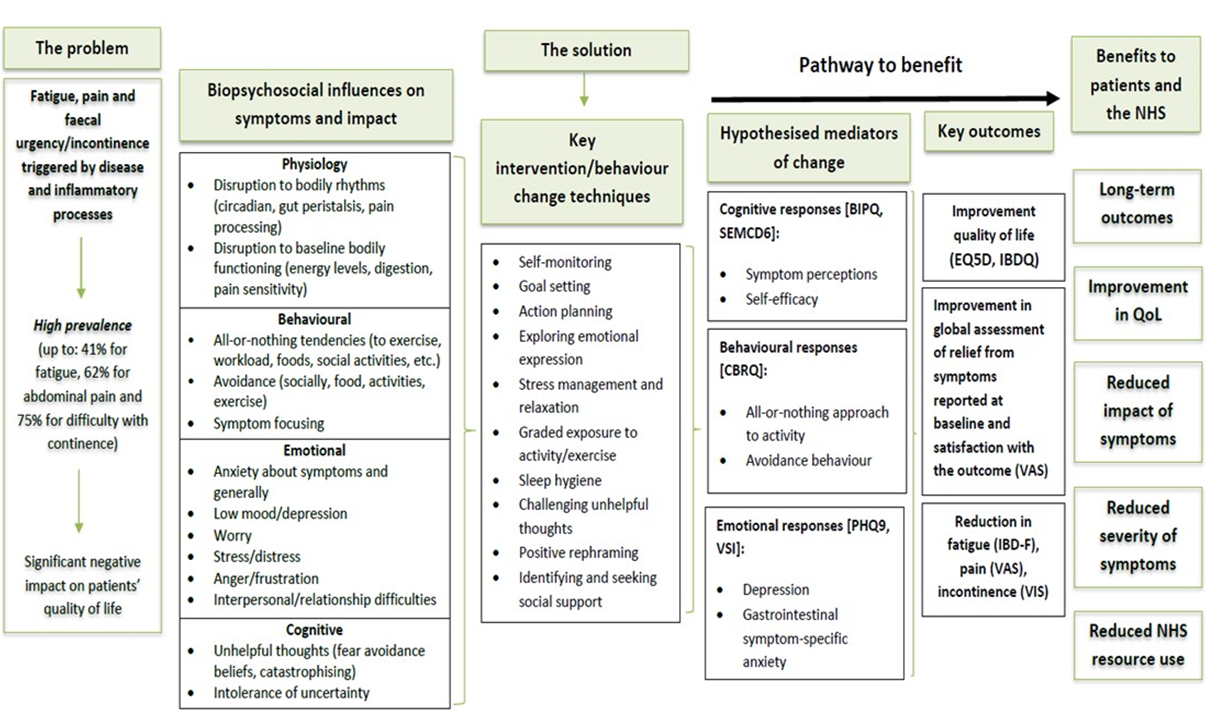

Supplement: Supplementary file 1 — Figure S1. [file BJHP-30-0-s001.docx]
