# Supplementary material for: Process evaluation findings contradict RCT results of the IBD‐BOOST digital self‐management intervention for fatigue, pain and faecal urgency in inflammatory bowel disease: A mixed methods study of patient perspectives
Source: Br J Health Psychol. 2025 Nov 14;30(4):e70035. doi: 10.1111/bjhp.70035 (PMC12617385; doi:10.1111/bjhp.70035)
Supplement: Supplementary file 2 — Table S1. [file BJHP-30-0-s003.docx]

**Table S1. IBD-BOOST programme content**

| **Core sessions** |  |
| --- | --- |
| 1. **Understanding your IBD symptoms**   *Tasks: complete individualised vicious cycle + symptom diary* | - Welcome to the programme - Factors that can contribute to fatigue, pain and urgency in IBD - Identifying factors that relate to you - Use of self-monitoring not symptom focusing - Setting your aims for the programme |
| 1. **Balancing your activity, eating and exercise**   *Tasks: reviewing goals for activity + sleep diary* | - Why are activity and exercise important? - Activity - Exercise - How fear leads to avoidance - Eating patterns - Setting your goals for activity and exercise |
| 1. **Improving your sleep**   *Task: reviewing goals for sleep* | - Why is sleep important? - Sleep habits - Sleep patterns - Improving your sleep - Setting your goals for sleep |
| 1. **Session 4a: Changing your thoughts: Part 1**   *Task: thought record* | - Why are thoughts important? - Identifying unhelpful thinking |
| 1. **Session 4b: Changing your thoughts: Part 2**   *Task: alternative thought record* | - Developing alternative thoughts |
| 1. **Session 5: Managing stress and coping with emotions**   *Tasks: reviewing goals for stress + stress diary* | - Why is managing stress and coping with emotions important? - The effects of stress and finding ways to manage it - The role of emotions and determining how best to take care of yourself - Setting your goals for managing stress and emotions |
| 1. **Session 6: Making the most of your social support and communication**   *Task: reviewing goals for social support* | - Types of social support - Communication and disclosure - Setting your goals for social support |
| **Symptom-specific sessions** |  |
| 1. **Session 7: Understanding fatigue in IBD** | - Types of fatigue - Factors related to IBD fatigue - Your vicious cycle of IBD fatigue - Practical strategies to manage fatigue |
| 1. **Session 8: Understanding pain in IBD** | - What is IBD-pain? - Acute and chronic pain in IBD: what’s the difference? - Causes of pain in IBD - A model of IBD pain - How can I best manage my pain? - Common questions around pain in IBD |
| 1. **Session 9: Managing urgency and leakage** | - Bowel functioning and bowel control difficulties - Stress and anxiety in urgency - Exercises to help reduce accidents - Practical bowel management tips - Using social networks to help manage urgency |
| 1. **Session 10: The role of acceptance and self-compassion in pain** | - What is acceptance and how can it help me? - Role of resilience - Practical exercises |
| **Summary session** |  |
| 1. **Session 11: Summary and maintaining improvement** | - Revisiting your programme aims - Preparing for the future - Sustaining and building upon improvements |
