# Supplementary material for: Process evaluation findings contradict RCT results of the IBD‐BOOST digital self‐management intervention for fatigue, pain and faecal urgency in inflammatory bowel disease: A mixed methods study of patient perspectives
Source: Br J Health Psychol. 2025 Nov 14;30(4):e70035. doi: 10.1111/bjhp.70035 (PMC12617385; doi:10.1111/bjhp.70035)
Supplement: Supplementary file 3 — Table S2. [file BJHP-30-0-s002.docx]

**Table S2. Topic guides**

1. **Topic guide for Process Evaluation: Pre-Intervention Patient Interviews**

| Opening question: | Can you tell me about your experience of IBD symptoms fatigue, abdominal pain, faecal urgency/incontinence (Time for this section no more than 10-12 minutes) |
| --- | --- |
| *Listen for and follow-up on any of these aspects:* | - *(To ask participant to confirm their age at last birthday/diagnosis)* - *Which of these symptoms are most bothersome for you?* - *Which of these symptoms affect your life most on daily basis?* - *In what way do they affect your life?* - *What aspects of your life are most affected?* - *When were you diagnosed with IBD? When did you start having symptoms of fatigue, abdominal pain, faecal urgency/incontinence?* - *Do you have any concerns in relation to the symptoms?* - *Do you have any other concerns?* - *Did you try any methods to manage the symptoms? What did you try? How effective were these methods?* |
| **Probing questions:** | **Did you talk to health professionals** (gastroenterologist, IBD nurses, dieticians, psychologists) **about your symptoms of fatigue, pain, faecal urgency/incontinence?** |
| *Listen for and follow-up on any of these aspects:* | - *Who did you talk to?* - *What information related to managing fatigue, abdominal pain, faecal urgency/incontinence did you receive?* - *Did you receive the advice that you needed? How useful was the advice?* - *Who else did you talk to about your symptoms? Other members of healthcare professionals, members of your family, friends, other patients?* - *How did you find the information in managing your symptoms?* |
| **Probing question:** | **What other sources do you get your information from about managing symptoms of fatigue, abdominal pain, faecal urgency/incontinence?** |
| *Listen for and follow-up on any of these aspects:* | - *Do you have any personal contacts with friends/family diagnosed with IBD who have symptoms of fatigue, abdominal pain, faecal urgency/incontinence?* - *Do you engage with a wider IBD community through e.g. social media / support groups for information about the symptoms?* - *What other sources have you accessed to get your information about symptoms of fatigue, abdominal pain, faecal urgency/incontinence IBD?* |
| **Probing question:** | **What do you feel the purpose of this study is?** |
| *Listen for and follow-up on any of these aspects:* | - What question do you feel this study is trying to answer? - [Provide research question to participant] How do you feel this study will answer this question. - By participating in this trial, you will be randomly allocated to one of two groups. What information have you had about what about the different groups? - Is there any more information you feel could be provided to better understand the purpose of this study? |
| **Probing question:** | **What information and support would help you with managing your symptoms?** |
| *Listen for and follow-up on any of these aspects:* | - *What are your reasons for volunteering to take part in this trial in relation to managing your symptoms?* - *What information and support would help you with managing your symptoms of fatigue, abdominal pain, faecal urgency/incontinence?* - *How (in what form e.g. verbal advice, factsheet, internet) should this programme be provided? What are your feelings/opinion about an online programme?* - *What are your feelings about CBT programmes?* - *What are your expectations and hopes from this trial about managing your symptoms of fatigue, abdominal pain, faecal urgency/incontinence?* - *What are your concerns about this trial in relation to your symptom management?* - *What do you expect to get out of the programme?* - *Do you have any particular expectations in terms of the structure and the content of the programme? What do you hope that the programme will cover?* - *Do you have any specific aspects in terms of the content and/or format that you hope that the programme will not include?* |
| **Closing question:** | **Is there anything else that you would like to add?** |

1. **Topic guide for Process Evaluation: Post-Intervention Patient Interviews – Intervention Group**

| **Opening question:** | **We met a few months ago – at the start of the trial. Can you tell me about your experience of IBD symptoms fatigue, abdominal pain, faecal urgency/incontinence since that time?** |  |
| --- | --- | --- |
| *Listen for and follow-up on any of these aspects:* | - *How are your symptoms now? Have they changed since our last meeting?* - *Which of the symptoms are most bothersome for you now?* - *What aspects of your life are most affected? Has that changed since you took part in the trial?* - *What bothers you in relation to your symptoms?* |  |
| **Probing question:** | **What do you think about the intervention for managing the symptoms of fatigue, abdominal pain, faecal urgency/incontinence?** |  |
| *Listen for and follow-up on any of these aspects:* | - *Tell me about any aspects of the intervention that you found helpful.* - *Tell me about any aspects of the intervention that you found unhelpful.* - *How effective were the methods to manage the symptoms of fatigue, abdominal pain, faecal urgency/incontinence?* - *What information related to managing fatigue, abdominal pain, faecal urgency/incontinence did you find most helpful?* - *Did you receive the information that you needed?* - *How useful was the information?* - *How effective were the methods of managing the symptoms?* - *Was the information relevant and useful to manage your symptoms?* |  |
| **Probing question:** | **Evaluating specific aspects and content of the intervention for managing symptoms of fatigue, abdominal pain, faecal urgency/incontinence** |  |
| *Listen for and follow-up on any of these aspects:* | - *What did you think about the seven core sessions?* - *What did you think about the specific sessions on fatigue, abdominal pain, faecal urgency/incontinence? (This would explore the session content, format and duration)* - *How often did you access the sessions? What affected the frequency of accessing the sessions? (e.g. structure or content of the session or factors related to the individual)* - *How long (on average) did it take you to complete each session? What do you think about the length of time needed to complete each session?* - *What was your preferred method of working through the sessions? (e.g. in one go or in small parts?)* - *Did you complete all the sessions? If not, what were the reasons for not completing the session(s)? If yes, what factors helped you in completing the sessions?*   *Did you do all the tasks set for each of the sessions? What did you think about the tasks?* |  |
| **Probing question:** | **What is your overall opinion about the intervention?** |  |
| *Listen for and follow-up on any of these aspects:* | - *Was the intervention helpful in terms of managing your symptoms?* - *Which strategies did you find most helpful for managing your symptoms? (To ask further questions on changing negative thinking, pacing, and other).* - *Which strategies did you find not useful for you?* - *Do you still use the strategies? How often do you use them and in what situations? (To explore use of strategies for each symptom reported).* - *What did you think about the format of the intervention? How did this relate to your expectations?* - *Did the intervention meet your expectations about managing your symptoms of fatigue, abdominal pain, faecal urgency/incontinence?* - *How did the intervention impact on your life? To explore possible positive and negative effects.* - *Would you recommend making any changes to the intervention? If yes, what changes would you recommend?* - *What did you think about the facilitator’s support? Did you find the phone calls/ emails useful or not? Please tell me more. (To ask further questions, depending on the initial response).* |  |
| **Closing question:** | **Is there anything else that you would like to add?** |  |

1. **Topic guide for Process Evaluation: Post-Intervention Patient Interviews – Control group**

| **Opening question:** | **We met a few months ago – at the start of the trial. Can you tell me about your experience of IBD symptoms fatigue, abdominal pain, faecal urgency/ incontinence since that time?** |  |
| --- | --- | --- |
| *Listen for and follow-up on any of these aspects:* | - *How are your symptoms now? Have they changed since our last meeting?* - *Which of the symptoms are most bothersome for you now?* - *What aspects of your life are most affected? Has that changed since you took part in the trial?* - *What bothers you in relation to your symptoms?* |  |
| **Probing question:** | **Did you do anything different in relation to your symptoms of fatigue, abdominal pain, faecal urgency/incontinence since you started the trial?** |  |
| *Listen for and follow-up on any of these aspects:* | - *Tell me if you have made any changes in relation to dealing with your symptoms of fatigue, abdominal pain, faecal urgency/incontinence? If yes, what changes did you make and why? Did the changes have any impact on your symptoms?* - *Did you try to find more information about your symptoms since you started the trial? What information did you find? What did you think about the information in relation to managing your symptoms?* - *Did you find any other methods to manage your symptoms of fatigue, abdominal pain, faecal urgency/incontinence? Did you try them? How effective were the methods?* |  |
| **Probing question:** | **Evaluating your satisfaction with being involved in the trial for managing symptoms of fatigue, abdominal pain, faecal urgency/incontinence** |  |
| *Listen for and follow-up on any of these aspects:* | - *How do you feel about being allocated to the control group? Tell me more about it.* - *What do you think the intervention may look like?* - *How do you think the intervention may have helped you?* - *What are your expectations from the intervention to managing symptoms of fatigue, abdominal pain, faecal urgency/incontinence?* - *How much time would you be prepared to allocate to the intervention?* - *Would you be prepared to do independent reading and homework as part of the intervention?* |  |
| **Probing question:** | **Do you intend to try the intervention when it is available at 12 months?** |  |
| *Listen for and follow-up on any of these aspects:* | - *Do you have any particular thoughts about the intervention?* - *What expectations of the intervention do you have* |  |
| **Closing question:** | **Is there anything else that you would like to add?** |  |
