# Supplementary material for: Process evaluation findings contradict RCT results of the IBD‐BOOST digital self‐management intervention for fatigue, pain and faecal urgency in inflammatory bowel disease: A mixed methods study of patient perspectives
Source: Br J Health Psychol. 2025 Nov 14;30(4):e70035. doi: 10.1111/bjhp.70035 (PMC12617385; doi:10.1111/bjhp.70035)
Supplement: Supplementary file 4 — Table S3. [file BJHP-30-0-s004.docx]

**Table S3. Qualitative data analysis process**

**Six-step process of data analysis and activities undertaken in each step**

| Steps of data analysis | Description of the activities |
| --- | --- |
| 1. Familiarisation with the data | Four researchers (WCD, SP, FA, and LD) and eight PPIE members conducted the data analysis. The researchers reviewed the transcripts multiple times to familiarise themselves with the data, noting initial ideas along the way |
| 1. Generating initial codes | The pre- and post-intervention transcripts were coded at different times, shortly after data collection. Initially, five transcripts from each set (pre- and post-interviews) were systematically coded line by line by four researchers independently, using an inductive approach. |
| 1. Searching for themes | The researchers met to reflect on the list of codes and discuss any differences in coding. An initial set of themes and sub-themes was developed for each dataset, providing a structure to guide the coding of the remaining transcripts. Two researchers then coded the remaining transcripts deductively using this framework. Three additional subthemes were identified. NVivo 14 software was used to support coding and data storage. |
| 1. Reviewing themes | To assess whether the themes aligned with the coded extracts, eight PPIE members were each provided with 6–8 transcripts and asked to code them using the existing framework. This process validated the thematic ‘map’ of the analysis. |
| 1. Defining and naming themes | Ongoing analysis was conducted to refine the specifics of each theme and the overall narrative of the findings, leading to the development of clear definitions and names for each theme. |
| 1. Producing the report | The final analysis and description, incorporating the selected extracts. Linking the findings back to the aims of the process evaluation and paper preparation for publication. |
